# Giant Solitary Fibrous Tumor of the Parotid Gland

**DOI:** 10.1155/2014/950712

**Published:** 2014-07-10

**Authors:** Octavian Chis, Silviu Albu

**Affiliations:** ^1^Oncology Institute “Prof. Dr. I. Chiricuţă”, Street Republicii No. 34-36, 400015 Cluj-Napoca, Romania; ^2^II-nd Department of Otolaryngology, Iuliu Hatieganu University of Medicine and Pharmacy Cluj-Napoca, Street Republicii No. 18, 400015 Cluj-Napoca, Romania

## Abstract

Solitary fibrous tumors (SFTs) are rare tumors that are mostly found arising from the pleura. SFT of the parotid gland is a rare tumor; only a few cases have been described in the literature. SFTs are benign in most cases. Clinically, SFTs usually manifest as well circumscribed, slow-growing, smooth, and painless masses. CT-Scan and MRI are the most sensitive imaging procedures used. The treatment of choice is complete surgical excision of the lesion. Since recurrence and metastasis can take place after several years, a lifelong clinical and imaging regular follow-up is compulsory. In this paper, we describe the diagnostic and therapeutic challenges of the up-to-now biggest parotid SFT. The clinical presentation, surgical management, and pathological and immunohistochemistry findings are described.

## 1. Introduction

Solitary fibrous tumor (SFT) was described by Klemperer and Rabin in 1931 as a tumor derived from the pleura [1]. Because of its alleged mesothelial origin, the tumor has been referred to by numerous other names (fibrous mesothelioma, localized fibrous tumor, localized mesothelioma, and benign mesothelioma), most of which are now outdated [2]. During the same period, Stout and Murray [3] defined hemangiopericytoma (HPC) as an uncommon vascular tumor, arising from perivascular cells known as pericytes, and arranged outside the basement membrane of the capillary wall. It was postulated that the pericytes possessed smooth muscle cell features and, thus, were responsible for vessel caliber regulation owing to their contractile capability, modulating both flux and permeability [4]. It was later demonstrated that SFT is most likely derived from adult mesenchymal stem cells, and its microscopic architecture and immunohistochemical characteristics make it nearly impossible to differentiate it from HPC [5].

Extrapleural SFTs have been reported in nearly all anatomic sites, with approximately 6% developing in the head and neck [6–8]. SFT of the parotid gland is a rare tumor; only a few cases been described in the literature [9–13]. In this paper we describe the diagnostic and therapeutic challenges of a giant parotid SFT.

## 2. Case Report

**Figure 1:**
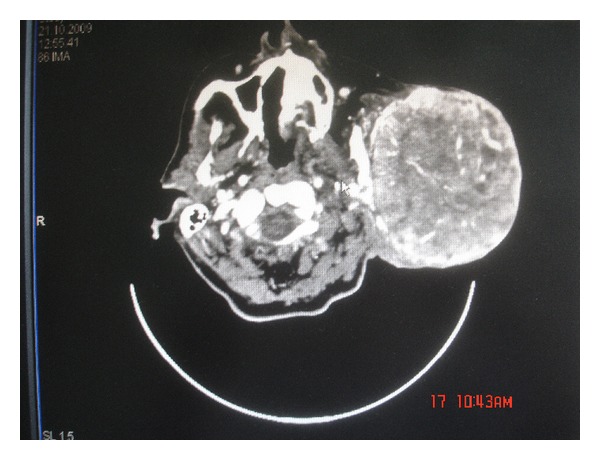
Postcontrast CT scan of the tumor.

**Figure 2:**
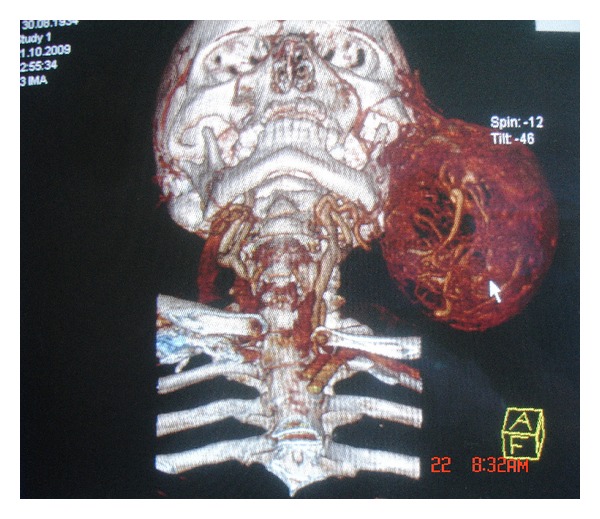
CT angiography displaying the rich vascular network.

A 67-year-old female presented with a 10-year history of a progressively developing, tender mass in the left parotid region. Her medical history was unremarkable. Physical examination revealed a 16 × 18 cm firm, fixed, immobile, smoothly contoured mass without overlying erythema. There were no facial palsy, no tumor in the pharynx and larynx, and no palpable lymph nodes, and the oral mucosa was intact. On the postcontrast computed tomography scan (Figure 1), a heterogeneously significantly enhancing mass with very large vascular structures within it is noticeable. It extended over the pretragal area and the left zygomatic arch, medially adjoining the mandible without associated bone destruction. There was no infiltration of the masticator space or the overlying skin. CT angiography demonstrated the rich vascular network (Figure 2) and preoperative embolization was performed. Considering the size of the mass, which had replaced the entire gland, a total parotidectomy was performed. The branches of the facial nerve were felt to be trapped within the tumor. To achieve complete resection, nerve stimulation was performed permitting conservation of the nerve, although resulting in a positive resection margin.

**Figure 3:**
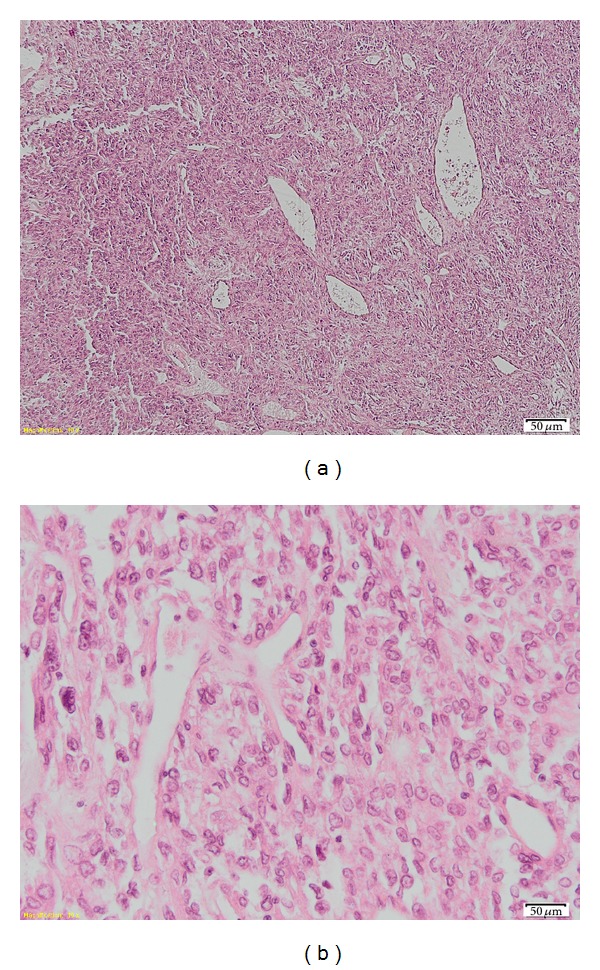
(a) Areas with heightened cellularity. The cells range from round/ovoid to slightly spindle; they are arranged randomly or in short ill-defined fascicles. (b) At higher magnification the tumor cells have indistinct cytoplasm and oval nuclei, usually with inconspicuous nucleoli.

**Figure 4:**
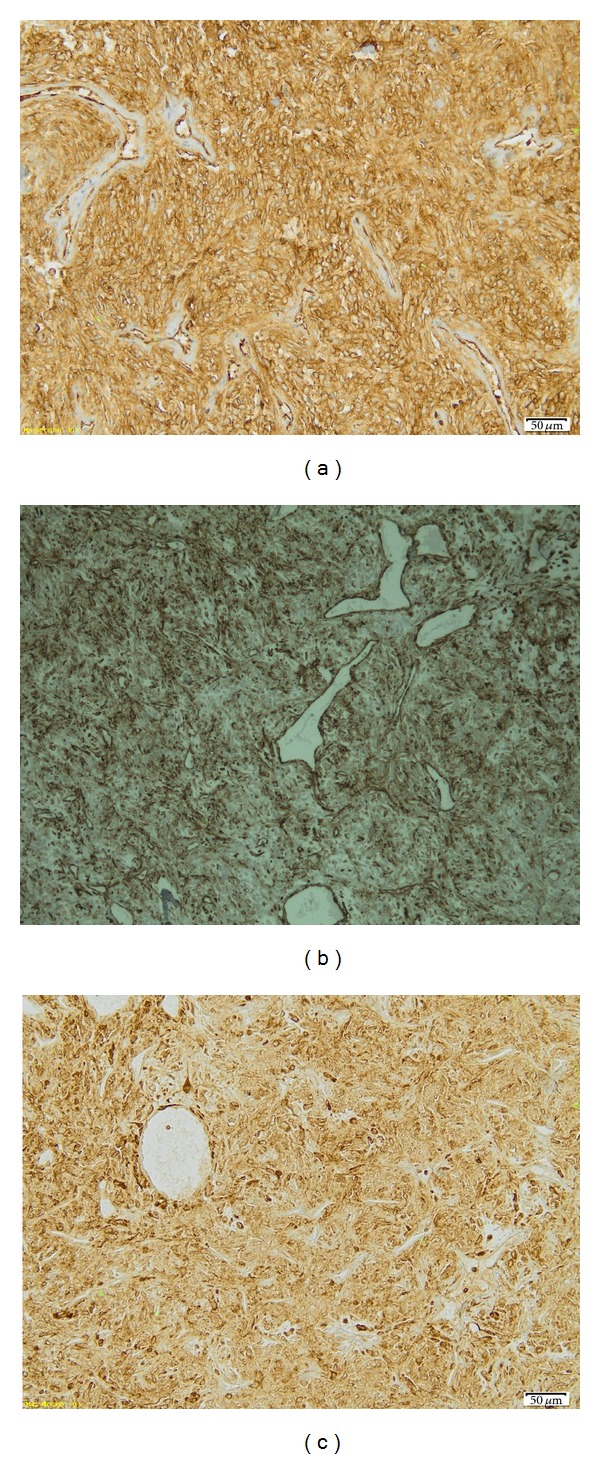
(a) A diffuse and strong cytoplasmic positivity of tumor cells is observed at this medium power magnification for CD34. (b) Vimentin immunohistochemical staining. (c) Strong cytoplasmic positivity for Bcl-2 is readily apparent.

Histology revealed alternating hypocellular and high cellular areas demonstrating a population of densely packed, randomly arranged cells (Figure 3(a)). The tumor cells are round to spindle with a predominantly fusiform appearance. Within the tumor, there were numerous thick-walled vessels with dilated vascular spaces, a HPC-like pattern. Mitosis was noted, the mitotic content being 4/10 high-power fields (hpf) (Figure 3(b)). Immunohistochemistry yielded positive for CD34 (Figure 4(a)), vimentin (Figure 4(b)), and bcl-2 (Figure 4(c)), yet negative for S 100, smooth muscle actin—SMA, and glial fibrillary acidic protein (GFAP). Based on the histology and immunohistochemistry report, a diagnosis of SFT was made.

## 3. Discussion

SFT is extremely rare in the parotid [8–13]. A recent review by Bauer et al. [14] identified only 22 cases in the literature. This is the biggest parotid fibrous tumor ever reported; previously described SFT in the parotid had a 12 cm diameter. There is a significant histological overlap between SFT and HPC. Gengler and Guillou [15] stated that most tumors in the past identified as HPC do not derive from pericytes but, instead, constitute a cellular variant of SFT. Thus, it was suggested to utilize the idiom “cellular SFT” to describe the nonpericytic HPCs and “fibrous SFT” to refer to the classic SFT. According to the World Health Organization Classification of Tumors, there is also overlap between SFT and giant cell angiofibroma [16]. However, this pattern is not recognized in the salivary gland.

According to the literature [9–14], parotid SFT is equally distributed between males and females, usually encountered in middle aged people, although it was described also in young patients. Patients present with a circumscribed, slowly growing, painless mass within the parotid. Occasionally, sleep apnea is reported, as a result of parapharyngeal extension of the parotid tumor [17].

Diagnostic work up includes CT and/or magnetic resonance (MR) imaging, even if results may not be specific for SFT. On CT scan, SFT appears as a well-defined soft-tissue mass relatively hyperdense with respect to adjacent tissues, demonstrating heterogeneous enhancement after contrast administration. On MRI, SFT has a signal characteristic consistent with any soft tissue tumor, with intermediate signal intensity on T1-weighted images and enhancement on T2-weighted images [9–14]. There are commonly heterogeneous bands within the tumor, perhaps as a result of the rich vascular supply. Angiography and preoperative embolization may be performed in cases of large tumors with significant vascular design [9–14].

Differential diagnosis is made with other enhancing lesions within the parotid gland, especially with pleomorphic adenomas and mucoepidermoid carcinomas. Large pleomorphic adenomas may have lobulated or poorly defined margins, while high grade mucoepidermoid carcinomas are scantily defined with heterogeneous internal architecture and may have associated cervical adenopathy [9–14].

Characteristically, SFT is a lobulated or nodular, firm, well-circumscribed, gray mass, surrounded by a pseudo-capsule, often with small satellite nodules separate from the main tumor [10–14]. Recently, fine-needle aspiration (FNA) has proved to be a key tool in diagnosing rare parotid masses [12, 14]. Unfortunately, due to lack of skilled workforce, we were not able to perform this investigation preoperatively. Definitive diagnosis is ascertained on histopathological and immunohistochemical analysis. The histological appearance of fibrous SFT is described by fibrous hypocellular areas alternating with hypercellular spots comprising round-to-spindle cells arranged in a fascicular, fibrosarcoma-like pattern. The occurrence of abundant and ramified vessels displaying thickened and hyalinized walls is a typical feature of fibrous SFT [9–14].

Histological features associated with malignancy include high cellularity, pleomorphism, necrosis, high mitotic rate (>6 mitoses/10 hpf in tumors considered malignant), and/or infiltrative margins [11–14]. However, Stout and Murray [3] did not notice any correlation between the mitotic activity and tumor behavior. They noted that the 10-year survival rates of patients with lesions that presented <4 mitosis/10 hpf, absence of necrosis and size below 6.5 cm were, respectively, 77%, 81%, and 92%. Alternatively, when the tumor displayed >4 mitosis/10 hpf, necrosis, and size greater than 6.5 cm, the ten-year survival rates were, respectively, 9%, 29%, and 63% [3]. Nevertheless, the histological appearance of SFT does not accurately predict a malignant behavior.

SFT shows immunoreactivity with vimentin and CD34, the major part of tumors displaying also positive results for bcl-2 and CD99. CD34 is the only consistently expressed and sensitive marker in SFT [11–14]. Absent reaction to S100, cytokeratin, SMA, desmin, muscle specific actin, smooth muscle myosin heavy chain, and GFAP is used to exclude other mesenchymal tumors [12–14].

Immunohistochemistry and histology help in the differential diagnosis from other parotid tumors: pleomorphic adenoma, myoepithelioma, fibrous histiocytoma, spindle cell squamous cell carcinoma, schwannoma, neurofibroma, fibrosarcoma, myofibroblastoma, meningioma, melanoma, Kaposi sarcoma, and synovial sarcoma [9–14].

Traditionally, the treatment of SFT has been surgical resection with negative margins [14]. Preoperative embolization may be employed in highly vascular tumors. Patients having complete tumor resection showed 100% survival at a mean 1.9 years follow-up [11]. However, according to the literature in cases of parotid gland SFT with positive margins, there have been no recurrences to date, although longer follow-up is required to make definite conclusions [9–14]. Since complete resection is the most important factor in clinical outcome, Cox et al. [18] stated that there is currently no evidence to support additional treatment beyond excision in malignant SFTs. Tumors that cannot be completely excised or which show malignant histological features may respond to radiation and/or chemotherapy. Since recurrence and metastasis can take place after several years, a lifelong clinical and imaging regular follow-up is compulsory [18].

## References

[1] Klemperer P, Rabin CB (1931). Primary neoplasms of the pleura: a report of five cases. *Archives of Pathology*.

[2] Chan JK (1997). Solitary fibrous tumour—everywhere, and a diagnosis in vogue. *Histopathology*.

[3] Stout AP, Murray MR (1942). Hemangiopericytoma: a vascular tumor featuring Zimmerman’s pericyte. *Annals of Surgery*.

[4] Billings KR, Fu YS, Calaterra TC (2000). Hemangiopericytoma of head and neck. *American Journal of Otolaryngology*.

[5] Rodríguez-Gil Y, González MA, Carcavilla CB, Santamaría JS (2009). Lines of cell differentiation in solitary fibrous tumor: an ultrastructural and immunohistochemical study of 10 cases. *Ultrastructural Pathology*.

[6] Gold JS, Antonescu CR, Hajdu C (2002). Clinicopathologic correlates of solitary fibrous tumors. *Cancer*.

[7] Ridder GJ, Kayser G, Teszler CB, Pfeiffer J (2007). Solitary fibrous tumors in the head and neck: new insights and implications for diagnosis and treatment. *Annals of Otology, Rhinology and Laryngology*.

[8] Brunnemann RB, Ro JY, Ordonez NG, Mooney J, El-Naggar AK, Ayala AG (1999). Extrapleural solitary fibrous tumor: a clinicopathologic study of 24 cases. *Modern Pathology*.

[9] Cho K, Ro JY, Choi J, Choi S, Nam SY, Kim SY (2008). Mesenchymal neoplasms of the major salivary glands: clinicopathological features of 18 cases. *European Archives of Oto-Rhino-Laryngology*.

[10] Muñoz Guerra MF, Amat CG, Campo FR, Pérez JS (2002). Solitary fibrous tumor of the parotid gland: a case report. *Oral Surgery, Oral Medicine, Oral Pathology, Oral Radiology, and Endodontics*.

[11] Mohammed K, Harbourne G, Walsh M, Royston D (2001). Solitary fibrous tumour of the parotid gland. *Journal of Laryngology and Otology*.

[12] Messa-Botero OA, Romero-Rojas AE, Chinchilla Olaya SI, Díaz-Pérez JA, Tapias-Vargas LF (2011). Primary malignant solitary fibrous tumor/hemangiopericytoma of the parotid gland. *Acta Otorrinolaringologica Espanola*.

[13] Suárez Roa MDL, Ruíz Godoy Rivera LM, Meneses García A, Granados-García M, Mosqueda Taylor A (2004). Solitary fibrous tumor of the parotid region. Report of a case and review of the Literature. *Medicina Oral*.

[14] Bauer JL, Miklos AZ, Thompson LDR (2012). Parotid gland solitary fibrous tumor: a case report and clinicopathologic
review of 22 cases from the literature. *Head and Neck Pathology*.

[15] Gengler C, Guillou L (2006). Solitary fibrous tumour and haemangiopericytoma: evolution of a concept. *Histopathology*.

[16] Fletcher CDM (2006). The evolving classification of soft tissue tumours: an update based on the new WHO classification. *Histopathology*.

[17] Sato J, Asakura K, Yokoyama Y, Satoh M (1998). Solitary fibrous tumor of the parotid gland extending to the parapharyngeal space. *European Archives of Oto-Rhino-Laryngology*.

[18] Cox DP, Daniels T, Jordan RC (2010). Solitary fibrous tumor of the head and neck. *Oral Surgery, Oral Medicine, Oral Pathology, Oral Radiology and Endodontology*.

